# Exploring the mechanism of YangXue QingNao Wan based on network pharmacology in the treatment of Alzheimer’s disease

**DOI:** 10.3389/fgene.2022.942203

**Published:** 2022-08-29

**Authors:** Yuying Zhang, Kaimin Guo, Pengfei Zhang, Mengying Zhang, Xiaoqiang Li, Shuiping Zhou, He Sun, Wenjia Wang, Hui Wang, Yunhui Hu

**Affiliations:** ^1^ Cloudphar Pharmaceuticals Co. Ltd., Shenzhen, China; ^2^ Tianjin Pharmaceutical and Cosmetic Evaluation and Inspection Center, Tianjin, China; ^3^ The State Key Laboratory of Core Technology in Innovative Chinese Medicine, Tasly Academy, Tasly Holding Group Co. Ltd., Tianjin, China; ^4^ Tasly Pharmaceutical Group Co. Ltd., Tianjin, China; ^5^ Key Laboratory of Molecular Biophysics, Hebei Province, Institute of Biophysics, School of Health Sciences and Biomedical Engineering, Hebei University of Technology, Tianjin, China; ^6^ Key Laboratory of Bioactive Materials Ministry of Education, School of Life Sciences, Nankai University, Tianjin, China

**Keywords:** YangXue QingNao Wan(YXQNW), Alzheimer’s disease, network pharmacology, cerebral blood flow (CBF), liver function, traditional Chinese medicine (TCM)

## Abstract

It is clinical reported that YangXue QingNao Wan (YXQNW) combined with donepezil can significantly improve the cognitive function of AD patients. However, the mechanism is not clear. A network pharmacology approach was employed to predict the protein targets and affected pathways of YXQNW in the treatment of AD. Based on random walk evaluation, the correlation between YXQNW and AD was calculated; while a variety of AD clinical approved Western drugs were compared. The targets of YXQNW were enriched and analyzed by using the TSEA platform and MetaCore. We proved that the overall correlation between YXQNW and AD is equivalent to clinical Western drugs, but the mechanism of action is very different. Firstly, YXQNW may promote cerebral blood flow velocity by regulating platelet aggregation and the vasoconstriction/relaxation signal pathway, which has been verified by clinical meta-analysis. Secondly, YXQNW may promote Aβ degradation in the liver by modulating the abnormal glucose and lipid metabolisms via the adiponectin-dependent pathway, RXR/PPAR-dependent lipid metabolism signal pathway, and fatty acid synthase activity signal pathway. We also verified whether YXQNW indeed promoted Aβ degradation in hepatic stellate cells. This work provides a novel scientific basis for the mechanism of YXQNW in the treatment of AD.

## 1 Introduction

Alzheimer’s disease (AD) is neurodegenerative disorder with progressive cognitive dysfunction in the elderly. With aging of the population, it is expected that there will be more than 100 million AD patients in the world by 2050. AD has caused severe distress for patients and has even become one of the most burdening diseases in the world (Scheltens et al., 2021). Cholinergic esterase inhibitors and NMDA receptor antagonists, as the first line of clinical drugs for AD, can only alleviate some symptoms of AD without changing AD progression (Masters et al., 2015). Because Alzheimer’s disease is a very complex disease, its etiology and pathology are still unclear. At present, the recognized pathological characteristics of AD are an accumulation of extracellular β-amyloids (Aβ), the formation of peptide plaques, and the hyperphosphorylation of tau protein (Lane et al., 2018). Most clinical trials of various drugs to clear Aβ plaque deposition in the brain have not been successful. Therefore, there is an urgent need for new treatments to prevent or slow down the process of AD. More and more experimental, epidemiological, and clinical evidence show that AD is not only a brain disease but is also a systemic disorder. Many peripheral systemic abnormalities are suggested to be involved in the pathological progression of AD (Wang J et al., 2017). Reduced cerebral blood flow has been reported in AD patients and contributes to AD progression (Mattsson et al., 2014; Kisler et al., 2017; Yew et al., 2017; Korte et al., 2020). Liver plays an essential role in the clearance of brain and plasma Aβ. It has been proved that plasma Aβ levels were positively correlated with impaired hepatic function and metabolic disorders (Estrada et al., 2019; Nho et al., 2019; Bassendine et al., 2020). Aiming at these peripheral abnormalities of AD may become a new strategy to prevent or slow down the process of AD.

There are research studies that suggest that the earliest event in AD is a decrease of cerebral blood flow (CBF). Cerebral blood flow reduction was found in both an AD patient, human-expressing ApoE4 protein which predisposes to AD, and a AD mice model, which contributes to the pathological progression of AD (Thambisetty et al., 2010; Michels et al., 2016; Korte, Nortley and Attwell 2020). Xueshuantong (XST), a TCM formula for increasing blood flow in humans in China, can improve learning and memory and motor performance in the APP/PS1 mice (Huang et al., 2018). These studies suggest that maintaining CBF may be an effective strategy for the treatment of AD in the future. In addition, more evidence suggesting new treatment strategy for AD may be targeted on liver function to decrease Aβ production and increase peripheral clearance (Estrada et al., 2019). As a first-line lipid-lowering drug, Atorvastatin has been shown to reduce AD risk possibility by upregulating liver LRP1 (Moon et al., 2011; Zissimopoulos et al., 2017). Pioglitazone, used for the treatment of type 2 diabetes, has been proved to promote Aβ clearance in the mice model. Two large phase III clinical trials of Pioglitazone in AD are ongoing (Mandrekar-Colucci et al., 2012; Galimberti and Scarpini 2017). Silymarin, a medicine herb for liver disease, has been reported to reduce the production of Aβ oligomers and has the potential for AD treatment (Murata et al., 2010).

Traditional Chinese medicine (TCM) has been widely used for hundreds of years to treat various chronic diseases including AD (Jarrell et al., 2018). YangXue QingNao Wan (YXQNW) is a traditional Chinese medicine produced by Tasly Pharmaceutical Co., Ltd. (Tianjin, China). It is composed of Dang Gui (Radix angelicae sinensis), Di Huang (Radix rehmanniae preparata), Chuan Xiong (Rhizoma chuanxiong), Bai Shao (Radix paeoniae alba), Ji Xue Teng (Caulis spatholobi), Yan Hu Suo (Rhizoma corydalis yanhusuo), Gou Teng (Ramulus uncariae cum uncis), Xia Ku Cao (Spica prunellae), Zhen Zhu Mu (Concha margaritifera usta), Jue Ming Zi (Semen cassiae), and Xi Xin (Herba asari). According to the TCM theory, YXQNW, which has the effect of tonifying blood and clearing liver heat, was widely used to treat diseases caused by blood deficiency and excessive liver yang, such as chronic cerebral circulation insufficiency (CCCI), headaches, dizziness, insomnia, and dreaminess, as well as dementia (Qu et al., 2016; Gu et al., 2018; Jia et al., 2020). Previous studies confirmed that YXQNW combined with donepezil can significantly improve the cognitive function and psychological abnormalities of AD patients (Cheng and Zang, 2013). Moreover, it is reported that YXQNW reduced the cognitive decline in APPswePS1dE9 transgenic mice (Wang X et al., 2017). However, by which pathway YXQNW alleviates cognitive decline in AD progression is unclear.

The present study aimed to explore the mechanism of YXQNW in the treatment of AD. We employed a network pharmacology approach to predict the active compounds of YXQNW, protein targets, and affected pathways in the treatment of AD based on network pharmacology and machine learning. Then, the enrichment analysis of the related pathways was carried out through the MetaCore database. Finally, the mechanism of “nourishing blood and calming liver” as suggested by the network analysis was further validated by the meta-analysis and hepatocyte experiment.

## 2 Materials and methods

### 2.1 Collection of drug’s targets

YXQNW-related literature studies published in the CNKI and PubMed databases were collected respectively. By August 2021, there were 1,257 articles from CNKI and 30 articles from PubMed on YXQNW. After reading and sorting out these literature studies, 47 genes regulated by YXQNW were recorded, which were defined as the literature targets of YXQNW. It is generally believed that the ingredients entering the blood are the important components in YXQNW. Additionally, in order to avoid missing critical components included in YXQNW, we selected the components of key herbs from detected components of YXQNW reported in the literature studies based on the HERB database (http://herb.ac.cn/). Finally, we got a list of 35 active ingredients of YXQNW (Supplementary Table S1). Three public databases were used to identify the active ingredients of YXQNW-related targets including HERB (http://herb.ac.cn/), ChEMBL (https://www.ebi.ac.uk/chembl/), and PubChem (https://pubchem.ncbi.nlm.nih.gov/). Combining the literature targets and active compound-related targets, we finally got 404 targets of YXQNW. The Drugbank, PubChem, STITCH, Swiss Target Prediction, and BATMAN-TCM databases were used to collect the targets of AD clinical Western medicine.

### 2.2 Collection of disease-related genes

The acknowledged disease-related targets were collected from the following 10 databases: GeneCards (https://www.genecards.org/), Open Targets (https://www.targetvalidation.org/), OMIM (https://omim.org/), DisGeNET (https://www.disgenet.org/), GWAS (https://www.ebi.ac.uk/gwas/), MalaCards (https://www.malacards.org/), HGNC (https://www.genenames.org/), Reactome (https://reactome.org/), MetaCore (https://portal.genego.com/), and KEGG (https://www.kegg.jp/). We collected genes related to different diseases including “Alzheimer’s disease”, “vascular dementia”, “hypertensive encephalopathy”, “hypertension”, “cerebral small vessel disease”, “cerebral small vessel diseases”, “prehypertension”, and “vertebrobasilar insufficiency” and artificially corrected them as disease-related gene sets. The genes collected in the database were artificially corrected as disease-related gene sets.

### 2.3 Correlation of drug targets and disease genes

To evaluate the efficacy of a drug on a disease, we adopted the method as described previously to study the correlation of the drug’s targets and the genes associated with the disease from the perspective of network propagation (Yang et al., 2020). Briefly, the drug targets and disease genes were respectively used as seed genes to run the Random Walk with Restart (RWR) algorithm (Kohler et al., 2008) in the background network STRING, which was performed in the R package dnet (version 1.1.7) with 0.75 % of the restart probability. In this way, we got the influence score vector of the two sets of seed nodes on all nodes in the background network, respectively. Pearson correlation coefficients of the two score vectors (Cor) were then calculated, and Z-score was used to evaluate the significance of the correlation.

$$\text{Z}‐\mathrm{score}=\frac{Cor-E\left(Cor\right)}{\delta \left(Cor\right)}$$

where E (Cor) and δ (Cor) are the mean and standard deviation of the Pearson correlation coefficients between the influence score vector of drug targets and those of 1,000 groups of random contrast disease genes, each of which contained the same number of randomly selected proteins as the disease seed nodes.

### 2.4 Drug similarity evaluation and hierarchical clustering based on the chemical structure, comprehensive target, and cellular function fingerprints

Generally, the similarity of two drugs is evaluated based mainly on the chemical structure, comprehensive targets, or cellular function fingerprints. Drug clusters with similar features are performed using hierarchical clustering. The chemical structure similarity is measured based on the Morgan fingerprint using RDKit in python. Molecular fingerprints encode the molecular structure in a series of binary digits (bits) that represent the presence or absence of particular substructures in the molecule. Comparing these fingerprints will enable us to determine the similarity between two molecules. Once SMILES strings are converted to scalar fingerprints, the Tanimoto coefficient is used as the similarity score to measure the absolute similarity between two molecules (Ozturk et al., 2016). The target similarity between two drugs is measured based on comprehensive targets of compounds. Two drugs acting on same targets could be considered to have the same effect. For multi-target drugs, drugs whose targets are very close in the PPI network show similar effects (Barabasi et al., 2011). Here, we used the network proximity index proposed by Barabasi et al. (Menche et al., 2015) to explore the similarity between two drugs. Briefly, the network proximity of drug-target module A and drug-target module B is defined using the separation measure as follows:

$$S_{AB}=d_{AB}-\frac{d_{AA}+d_{BB}}{2}$$

which compares the mean shortest distance within the interactome of each target modules, ⟨*d*_*AA*_⟩ and ⟨*d*_*BB*_⟩, to the mean shortest distance ⟨*d*_*AB*_⟩ between the target modules A and B. The smaller the *S*_*AB*_, the closer the topological distance between the two drugs, that is, the more similar the functions of the two drugs. BNet was used as the background human PPI network, which excluded inferred data, such as evolutionary analysis, gene expression data, and metabolic associations, and contains 16,667 nodes and 243,603 edges (Yang, Tian, Zhao and Zhang 2020).

The cellular function fingerprint similarity of the two drugs was measured based on a “compound-target–cellular function” heterogeneous network using the PathSim method (Sun et al., 2011). As described in the literature (Guo et al., 2020), in the “compound-target–cellular function” heterogeneous network, the metapath “compound-target–cellular function-target–compound” of the two drugs was considered to describe the linkage between the two drugs. In this instance, cellular function fingerprints of compounds were described using GO biological processes term of targets of compounds. Under the metapath framework, PathSim was developed to find peer objects in the network and to measure the similarity of the peer objects based on metapaths. The “compound-target–cellular function” heterogeneous network consists of drug-comprehensive target interactions and target–cellular function relations from the Gene Ontology database (Carbon et al., 2017). Given a set of N compounds to be clustered, an N × N similarity matrix was generated. Finally, the similarity matrix was used to perform a hierarchical cluster, which was executed by the R package hClust.

### 2.5 Differential gene expression analysis for Alzheimer’s disease

A total of six transcriptomics datasets were collected from two types of transgenic AD mouse models including HO-TASTPM and APP/PS1, consisting of samples with three different brain regions, including the hippocampus, frontal cortex, and brain, and two time points (mouse age: 8 and ∼18 months). All types of transgenic AD mouse models were manipulated to result in AD-related gene mutations, such as APP mutants, which can develop Aβ aggregation or neurofibrillary tangles (NFTs), resulting in cognitive impairment.

The brain microarray datasets were obtained from the Gene Expression Omnibus (https://www.ncbi.nlm.nih.gov/geo), including GSE64398 (Cummings et al., 2015; Matarin et al., 2015), GSE65067 (Wang et al., 2015; Ulland et al., 2017), and GSE74615 (Orre et al., 2014), according to the different mouse ages and brain regions. Differential expression analysis was performed with GEO2R. Briefly, all raw expression data were log2 transformed, and all samples were quantile-normalized together. Probe IDs in each dataset were mapped to NCBI entrez IDs and probes mapping to multiple genome regions or without their corresponding entrez IDs were removed. The obtained gene data were then used to conduct the differential expression analysis using the LIMMA package (Ritchie et al., 2015). The genes with FDR<0.05 and |log2FC|>1.5 were identified as differentially expressed genes (DEGs). Finally, all DEGs were converted into unique human-orthologous genes using the Mouse Genome Informatics (MGI) database (http://www.informatics.jax.org/).

### 2.6 Pathway enrichment analysis

The GO and KEGG enrichment analyses were performed using DAVID (https://david.abcc.ncifcrf.gov/). Gene Set Enrichment Analysis was performed using the Gene Set Enrichment Analysis (GSEA) software from Broad Institute (Mootha et al., 2003; Subramanian et al., 2005). Tissue Specific Expression Analysis (TSEA) was performed using the TSEA tool website (http://genetics.wustl.edu/jdlab/tsea/). The MetaCore Database Analysis Platform (https://portal.genego.com/) was used for pathway enrichment through the enrichment function of the Pathway Maps of YXQNW target sets.

### 2.7 Meta-analysis

Four mainstream medical databases were searched, including PubMed, CNKI, WANFANG, and VIP. The main content is a randomized controlled trial (RCT) of YXQNW combined with conventional treatment in the treatment of cardiovascular diseases. The time frame used for database queries was from the earliest indexed studies to July 2021. Search words included “Yangxue Qingnao”. RCTs of YXQNW and/or combined with conventional therapy in the treatment of cardiovascular diseases. Patients diagnosed with a cardiovascular disease and treated with YXQNW or combined with conventional therapy. The basic treatment of the control group was the same as that of the experimental group, and the experimental group was treated with YXQNW on the basic treatment. Hemodynamic indexes: Blood flow velocity of vertebral artery, basilar artery, anterior cerebral artery, posterior cerebral artery, posterior cerebral artery, and clinical efficacy. The following studies were excluded from the meta-study: duplicate studies; non-randomized trials; case reports, reviews, systematic reviews, and abstracts. The basic treatment of the control group and the experimental group was inconsistent. Two researchers selected the articles independently. First, Endnote was used for screening according to the preset criteria, with repeated literature in the databases excluded. Then, the researchers read the titles and abstracts to exclude clearly irrelevant literature. Finally, the remaining studies were read in full, finalizing the literature selection based on the inclusion and exclusion criteria. After selection, the two researchers independently extracted the data from the included studies using Excel, including: 1) the basic information of the first author’s name and publication date; 2) sample size, participants’ mean age and gender, duration of treatment, interventions in the experimental and control groups, and prognostic indexes. The quality of the included studies was assessed in the Cochrane Handbook for Systematic Reviews of Interventions. The assessment items included random allocation, allocation concealment, and blinding. The included literature research data were statistically analyzed by using the Revman5.3 software. Q test and I2 value were used to analyze the heterogeneity among the included studies. Mean difference (MD), odds ratio (OR), and 95% CI were used as the analysis statistics, *p* ≤ 0.05 is considered for the study to be statistically significant. *p* > 0.1 and I^2^ < 50 % indicated a good consistency or lower heterogeneity among the included studies, and a fixed effect model was adopted; I^2^ ≥ 50% indicated high heterogeneity between the results of the study, and a random effect model was adopted.

### 2.8 Aβ accumulation in hepatic stellate cell

Rat hepatic stellate cell HST-T6 was cultured in DMEM + 10% FBS. HST-T6 cells were inoculated in a 24-well culture plate. The cells were divided into groups, and the doses of YXQNW in different groups were given as 0.025 mg/ml, 0.05 mg/ml, and 0.2 mg/ml. Memantine (100 μM, S2043, Selleck company, Shanghai, China) was used as negative control drug. After 48 h of incubation, the cell was gently washed with PBS and incubated at 37°C for 20 min. The PBS was discarded and Aβ_1-42_ was added (1 μM, A118755, Aladdin Company, Shanghai, China)into the cell except blank control group. The cold PBS was used to terminate the ingestion of Aβ_1-42_ after 1 h of ingestion. The cells were washed with PBS thrice and then lysed with a lysate. Cellular Aβ accumulation was detected by an Aβ ELISA kit (J10414, JingMei Company, Jiangsu province, China). One-way ANOVA and appropriate post-hoc analyses were used for comparisons of more than two groups. *p* < 0.05 was considered statistically significant.

## 3 Results

### 3.1 The efficacy of YangXue QingNao Wan on Alzheimer’s disease by network analysis

**FIGURE 1:**
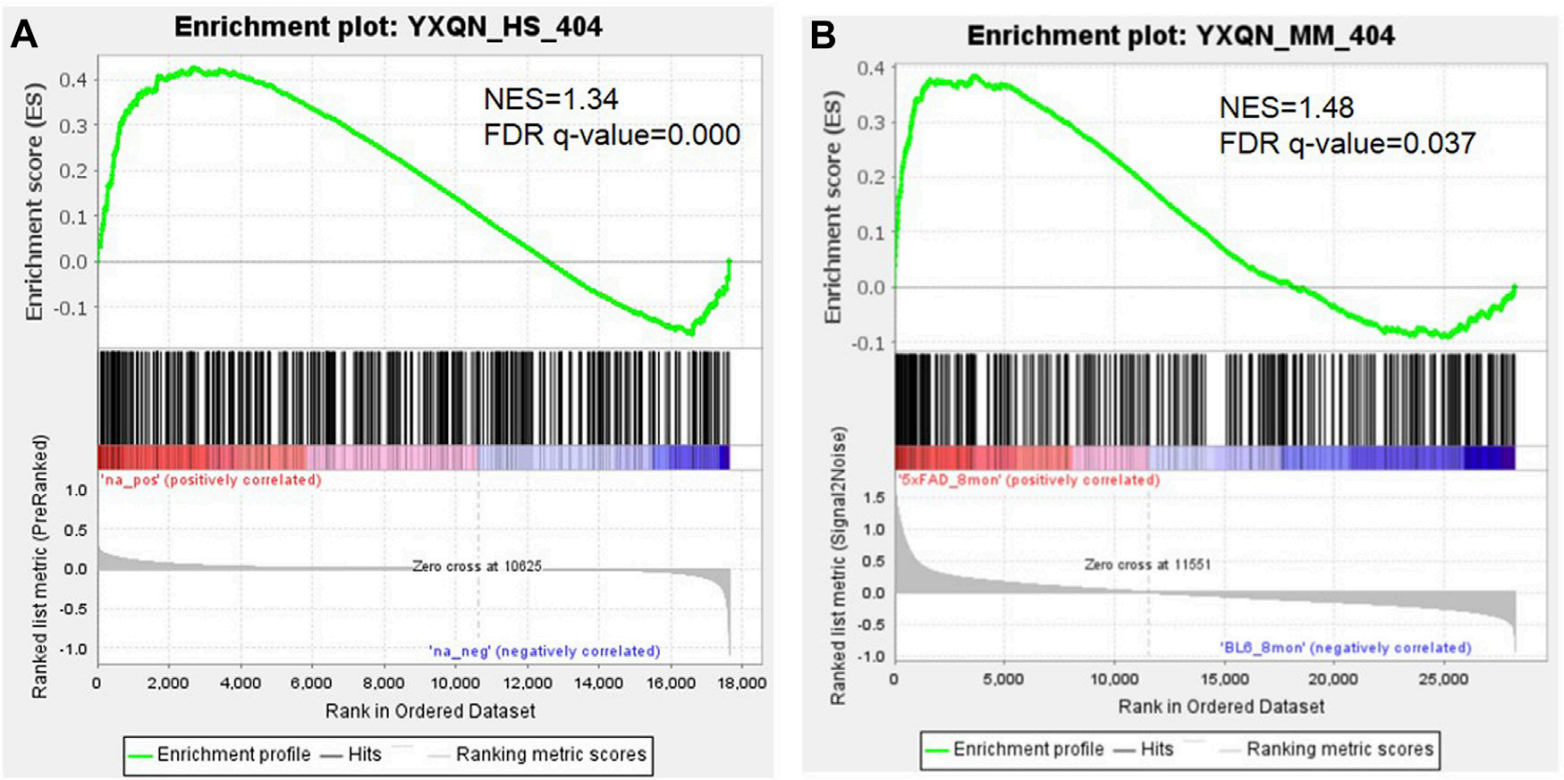
The GSEA analysis of YXQNW targets with transcriptome data of the AD patient **(A)** and AD mice model **(B)**.

The text continues here. At present, it is reported that YXQNW may be effective for a variety of indications including Alzheimer’s disease, vascular dementia, hypertensive encephalopathy, hypertension, cerebral small vessel diseases, prehypertension, and vertebrobasilar insufficiency. In order to evaluate the efficacy of YXQNW on AD and its related indications, the correlation of YXQNW’s targets and disease-related genes were analyzed by the network according to the method of previous literature studies (Yang, Tian, Zhao and Zhang 2020). Supplementary Table S2 shows that the YXQNW’s targets were positively correlated with the disease-related genes on the network, and all correlations were significant (Z-score >3). These results indicate that YXQNW was indeed effective for the aforementioned indications, especially Alzheimer’s disease, at a molecular network level. We identified AD gene sets based on transcriptome data from the blood of the AD patient (GSE63060) (Sood et al., 2015) and the cortex of the 8 month-old AD mice model (GSE168137) (Forner et al., 2021) using the Gene Set Enrichment Analysis (GSEA) method. The GSEA results revealed that the targets of YXQNW were significantly positive enriched from the AD-related gene, whether the AD gene set is from the blood of AD patients (NES = 1.34, FDR q value < 0.001, Figure 1A) or the cortex of 8 month-old 5xFAD mice models (NES = 1.48, FDR q value = 0.037, Figure 1B). Meanwhile, the transcriptome data from the frontal cortex tissue of AD patients (GSE185909) (Beck et al., 2022) were also used as ranked gene sets to inspect the enrichment of the targets of YXQNW, and the result showed that there was a same trend as that of the cortex tissue from the mice models (NES = 1.22, FDR q value = 0.17, Supplementary Figure S1).

Meanwhile, we also calculated the correlation of targets of YXQNW with a multi-dataset of AD-related DEGs, respectively, which were archived from different brain regions, including the hippocampus, frontal cortex, and brain of two ages of the transgenic mouse model (Supplementary Table S3). The results showed that each of the AD-related DEG sets had a significant correlation with the targets of YXQNW (Z-score > 3), suggesting that YXQNW may work as an effective treatment of AD (Supplementary Table S4, Supplementary Figure S2).

### 3.2 The comparison between YangXue QingNao Wan and clinical Western medicine on Alzheimer’s disease by network analysis

We further selected some FDA-proved drugs for AD as control including haloperidol, donepezil, memantine, risperidone, trifluperidol, galantamine, piracetam, quetiapine, rivastigmine, citalopram, vinpocetine, and idebenone. The correlation of the aforementioned 12 clinical Western medicines’ targets and disease-related genes were analyzed based on the network. Supplementary Table S5 shows that all the 12 clinical Western medicine targets were positively correlated with the disease-related genes on the network, and all correlations were significant (Z-score > 3). The results also indicated that the overall correlation between YXQNW and AD is equivalent to AD clinical Western drugs. Moreover, we tested the correlation between AD and two unrelated drugs (Pegvaliase and Sapropterin, clinical drugs for Phenylketonuria) as negative control. The results showed that there was no significant correlation between the negative drugs and AD (Z-score < 3), indicating the reliability of this method.

**FIGURE 2:**
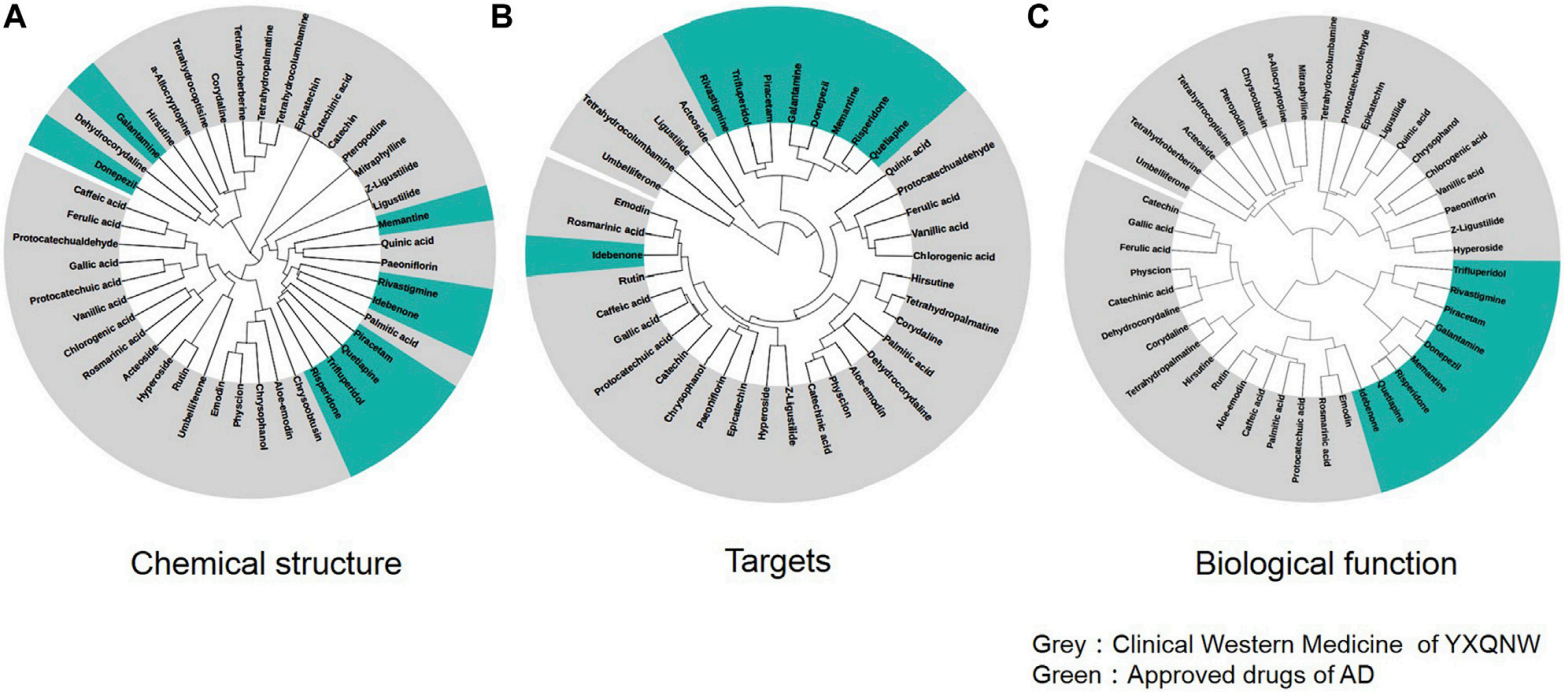
Unsupervised hierarchical clustering of YXQNW compounds and approved drugs based on the similarity of the chemical structure **(A)**, drug comprehensive targets **(B)**, and biological functions **(C)**.

We selected 9 clinical drugs of AD as control with different MOAs, including an AChE inhibitor (donepezil, galantamine, and rivastigmine), serotonin, and dopamine receptor antagonists (quetiapine, trifluperidol, and risperidone), NMDA receptor antagonist (memantine), neurotransmitter GABA regulator (piracetam), and antioxidant (Idebenone). As mentioned in the methods section, 35 compounds were selected as the active compounds of YXQNW. Firstly, we studied the chemical structural similarity between YXQNW compounds and the nine kinds of AD drugs. Figure 2A shows the hierarchical clustering of chemical compounds based on structural similarity of YXQNW active compounds and AD clinical drugs. The results revealed that most of the active compounds of YXQNW did not cluster with the 9 clinical AD drugs, which indicates that the YXQNW active compounds did not have a similar structure with clinical AD drugs (Figure 2A). Moreover, the hierarchical clustering of the compounds based on target similarity showed that AD clinical drugs did not cluster with most YXQNW-active compounds, which indicates that YXQNW did not have the same targets with clinical AD drugs (Figure 2B). Unsupervised hierarchical clustering of compounds based on biological functional similarity also showed that YXQNW-active compounds did not cluster with the 9 clinical AD drugs (Figure 2C). All results displayed that YXQNW shares no similar chemical structures, targets, and cellular functions with AD clinical drugs by unsupervised learning, hierarchical clustering.

### 3.3 Tissue enrichment and pathway enrichment of the YangXue QingNao Wan compound targets

**FIGURE 3:**
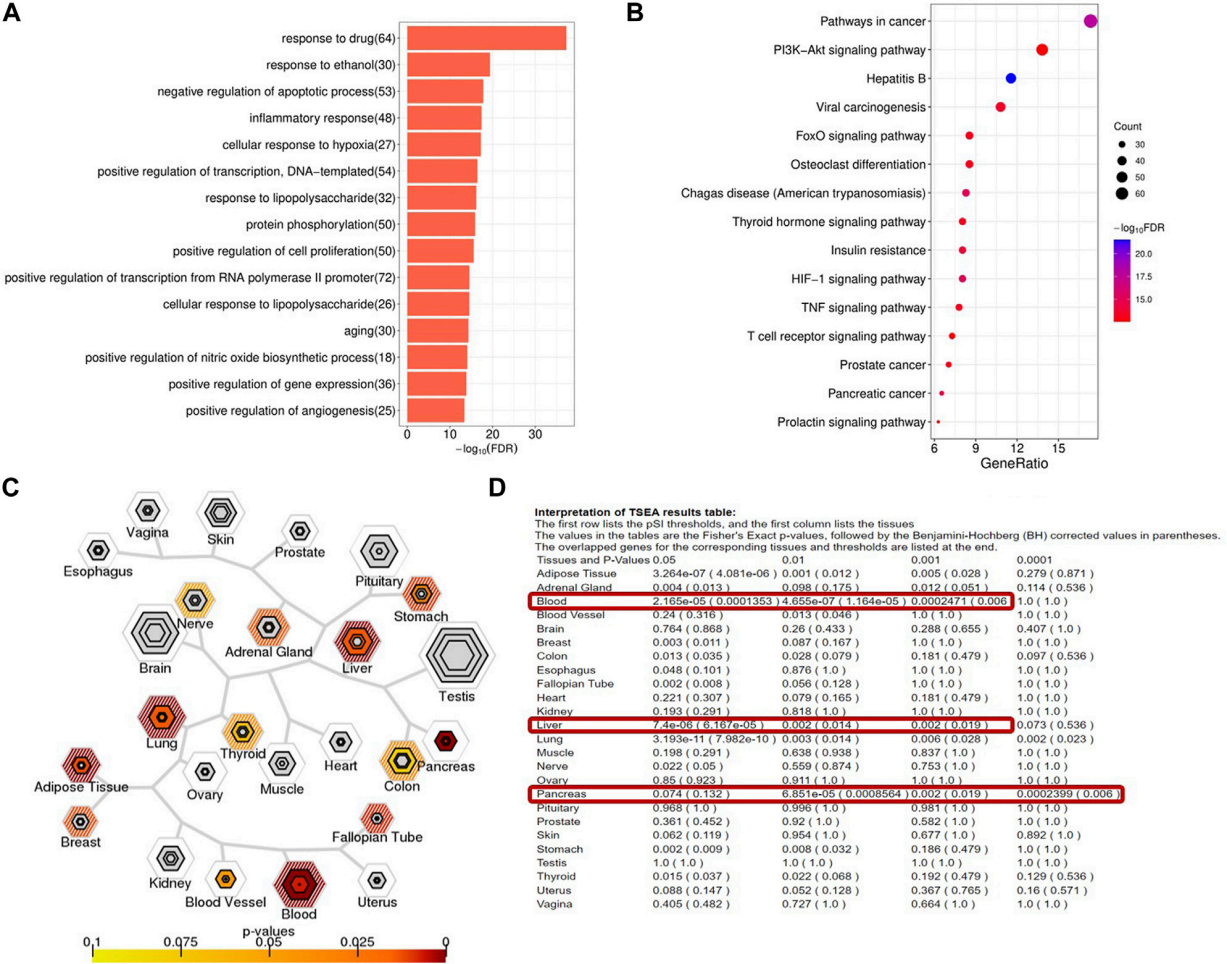
Functional enrichment and tissue enrichment of the YXQNW compound targets. **(A)** GO analysis; **(B)** KEGG analysis; **(C)** TSEA analysis; **(D)** tissue enrichment.

We first analyzed the targets of YXQNW by using the GO and KEGG analyses. The GO analysis results showed that targets of YXQNW enriched in some AD-related biological processes such as “negative regulation of apoptotic process” (*p* = 1.42E-21), “inflammatory response” (*p* = 4.81E-21), “aging” (*p* = 1.27E-17), and “positive regulation of nitric oxide biosynthetic process” (*p* = 3.42E-17) (Figure 3A). The KEGG analysis results showed that targets of YXQNW enriched in some AD-related pathway such as the “PI3K-Akt signaling pathway” (*p* = 3.48E-16), “insulin resistance” (*p* = 7.12E-16), and “TNF signaling pathway” (*p* = 4.20E-15) (Figure 3B), which have been reported to be associated with AD by different groups (Decourt et al., 2017; Kellar and Craft 2020; Wang et al., 2020). To further explain the mechanism of YXQNW in AD treatment, we performed tissue enrichment (Figures 3C, D) with the YXQNW targets. The results showed that the targets of YXQNW were significantly enriched in blood, liver, pancreas, and adipose tissues but not in the brain. Unlike the current clinical AD drugs directly acting on the brain, this result indicates that the therapeutic effect of YXQNW on AD is likely to go through the peripheral system, especially targeting the blood and liver.

### 3.4 YangXue QingNao Wan may promote blood flow velocity in Alzheimer’s disease treatment

**FIGURE 4:**
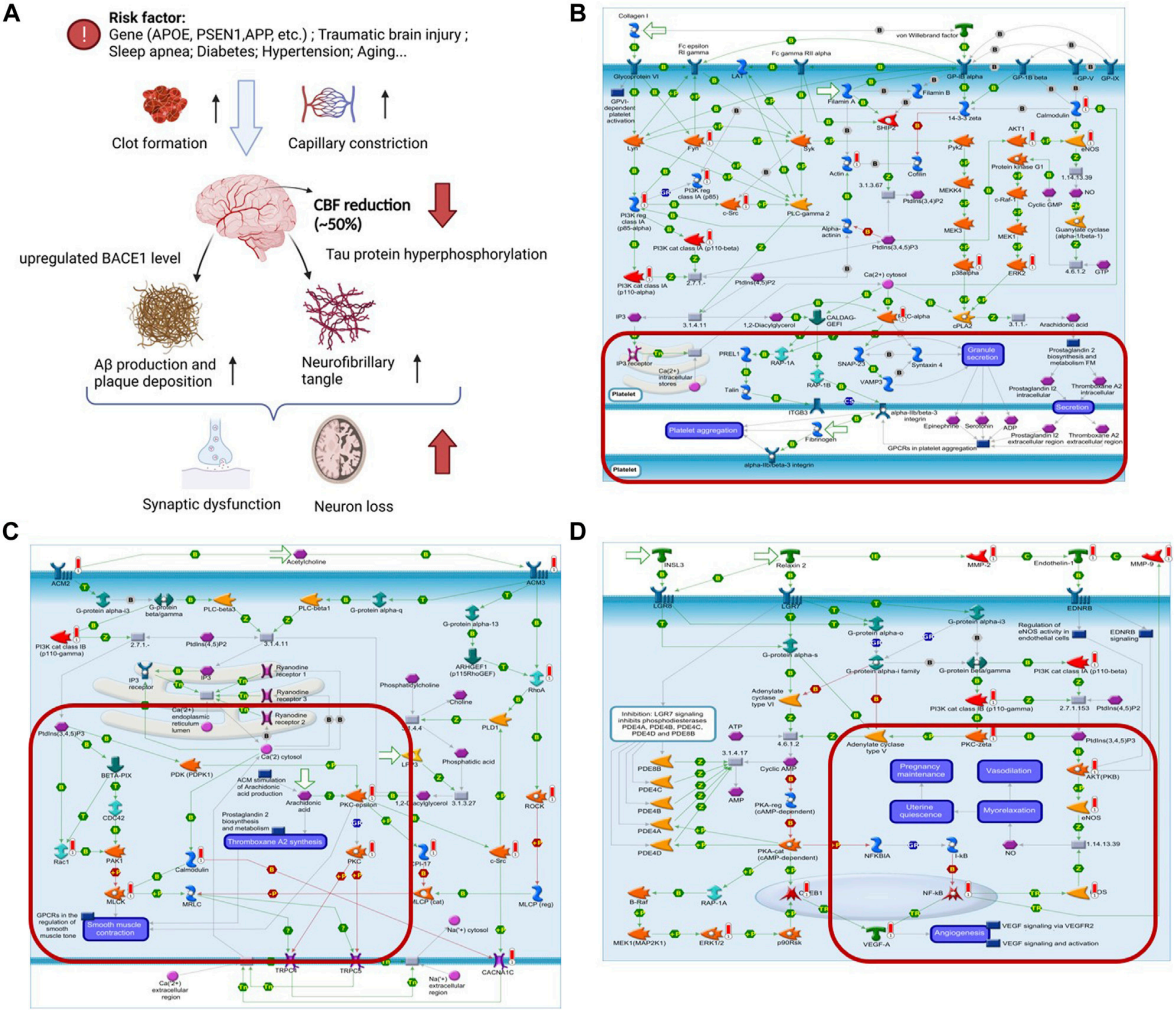
YXQNW may promote blood flow velocity in AD treatment. **(A)** Summary of CBF reduction in AD; **(B)** regulation of blood coagulation_GPIb-IX-V-dependent platelet activation; **(C)** ACM regulation of smooth muscle contraction; **(D)** muscle contraction and vasodilation_relaxin signaling pathway. (The red marker is the target of YXQNW).

CBF is reduced in AD possibility by capillaries constriction, neutrophil trapping in the capillaries, and clot formation (Korte, Nortley and Attwell 2020). The reduction of CBF accelerates neuron loss by upregulating the BACE1 enzyme that makes Aβ accumulation (Guo, Tang, Zhang, Wei, Tang, Wu and Yang 2020). Therefore, CBF reduction may play an important role in driving the cognitive decline by initiating the amyloid cascade or amplifying Aβ production (Figure 4A). Many clinical evidence and animal experiments have shown that YXQNW can improve CBF. The YXQNW target set was analyzed for pathway enrichment through the enrichment function of the Pathway Maps in the MetaCore Database Analysis Platform. We focused on the pathway maps related to the regulation of blood flow velocity. The results showed that YXQNW may regulate the blood flow velocity through the following three pathways: 1) Regulation of blood coagulation_GPIb-IX-V-dependent platelet activation (Figure 4B); 2) ACM regulation of smooth muscle contraction (Figure 4C); and 3) muscle contraction and vasodilation_relaxin signaling pathway (Figure 4D). In addition, YXQNW may promote the blood flow velocity by GPCR-dependent platelet aggregation and eNOS activity dependent smooth muscle relaxation (Data not shown).

**FIGURE 5:**
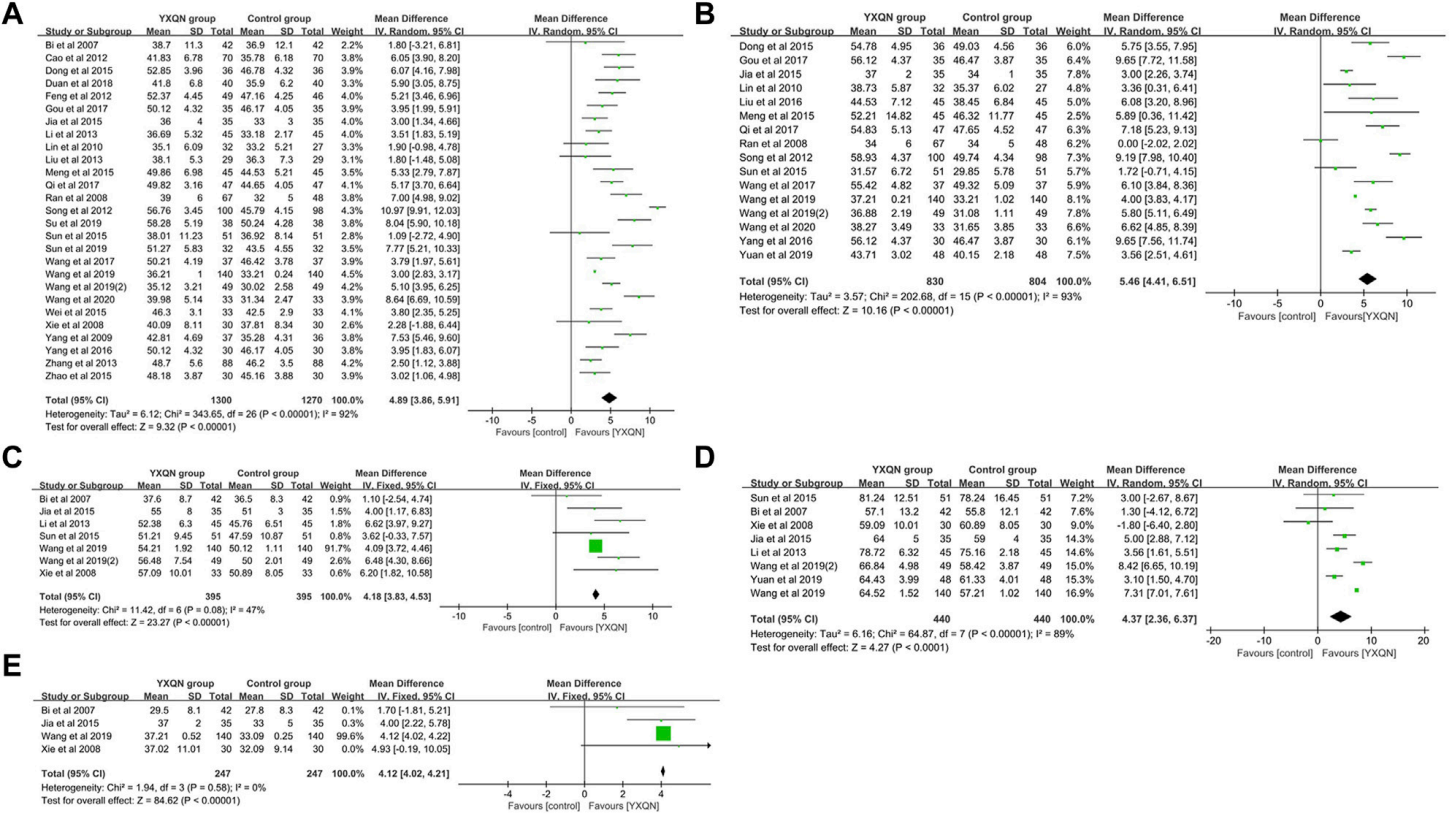
Meta-analysis of YXQNW on **(A)** blood flow regulation of the basilar artery; **(B)** vertebral artery’s blood flow velocity; **(C)** anterior cerebral artery’s blood flow velocity; **(D)** middle cerebral artery’s blood flow velocity; and **(E)** posterior cerebral artery blood flow velocity.

A total of 1,215 relevant literature studies were obtained from the aforementioned Chinese and English databases. After reading the title, abstract, and full text, 30 clinical studies were finally included. A total of 2,784 participants, including 1,384 in the experimental group and 1,364 in the control group. The screening process is shown in Supplementary Figure S3. Supplementary Figures S4, S5 show summaries of the quality and bias risks of the included studies. A total of 30 clinical study data were included to evaluate the changes of clinical efficacy before and after treatment. There was homogeneity among the studies (*p* = 0.74, I^2^ = 0). The calculation and analysis results of the fixed effects model showed that the difference of clinical efficacy was statistically significant [RR = 1.25%, 95% CI (1.20, 1.29), *p* < 0.000 01], indicating that YXQNW can significantly improve the clinical efficacy of cardiovascular diseases, as shown in Supplementary Figure S6. A total of 27 studies were included to evaluate the changes of basilar artery blood flow velocity before and after YXQNW treatment of cardiovascular diseases. A meta-analysis was carried out by using the random effects model (*p* < 0.000 01, I^2^ = 92%). The results showed that, compared with the routine treatment of cardiovascular diseases, the acceleration of the basilar artery blood flow velocity after YXQNW was greater [MD = 4.89%, 95% CI (3.86, 5.91), *p* < 0.000 01] (Figure 5A). A total of 16 clinical research data were included to evaluate the changes of the vertebral artery blood flow velocity before and after YXQNW treatment of cardiovascular diseases. Heterogeneity test *p* < 0.000 01, I^2^ = 93%. The results of the meta-analysis of the random effects model showed that YXQNW combined with routine treatment could significantly accelerate the blood flow velocity of the vertebral artery, and the difference was statistically significant [MD = 5.46%, 95% CI (4.41, 6.51), *p* < 0.000 01], as shown in Figure 5B. A total of seven studies were included to evaluate the changes of blood flow velocity of the anterior cerebral artery before and after treatment. The heterogeneity test *p* = 0.08, I^2^ = 47%. The meta-analysis results of the fixed effects model showed that YXQNW combined with routine treatment was 4.18 times higher than that of routine treatment alone, and the difference was statistically significant [MD = 4.18%, 95% CI (3.83, 4.53), *p* < 0.000 01], see Figure 5C. Eight studies were included to evaluate the changes of blood flow velocity of the middle cerebral artery before and after YXQNW treatment of cardiovascular diseases. The random effects model was used for meta-analysis, and the results (*p* < 0.00001, I^2^ = 89%) showed that the difference was statistically significant [MD = 4.37%, 95% CI (2.36, 6.37), *p* < 0.0001]. The meta-analysis results of the random effects model showed that compound YXQNW when combined with routine treatment could significantly accelerate the blood flow velocity of the middle cerebral artery, as shown in Figure 5D. Four studies were included to evaluate the changes of the blood flow velocity of the posterior cerebral artery before and after treatment. There was homogeneity among the studies (*p* = 0.58, I^2^ = 0%). The results of the fixed effects model showed that compared with the routine treatment of cardiovascular diseases, the acceleration of the blood flow velocity of the basilar artery after YXQNW combined with routine treatment was greater, and the difference was statistically significant [MD = 4.12%, 95% CI (4.02, 4.21), *p* < 0.0001], as shown in Figure 5E. To analyze the publication bias of the clinical efficacy of YXQNW in the treatment of cardiovascular diseases, the results show that the research literature is evenly distributed around the vertical line, suggesting that the possibility of publication bias is small, as shown in Supplementary Figure S6. Consistent with our previous hypothesis, the results of clinical meta-analysis verified the up-regulation effect of YXQNW on cerebral blood flow velocity, suggesting that YXQNW may increase the CBF of AD patients by increasing the blood flow velocity of the basilar artery, vertebral artery, anterior cerebral artery, middle cerebral artery, and posterior cerebral artery.

### 3.5 YangXue QingNao Wan promotes Aβ degradation by regulating the glucose and lipid metabolisms in the liver

**FIGURE 6:**
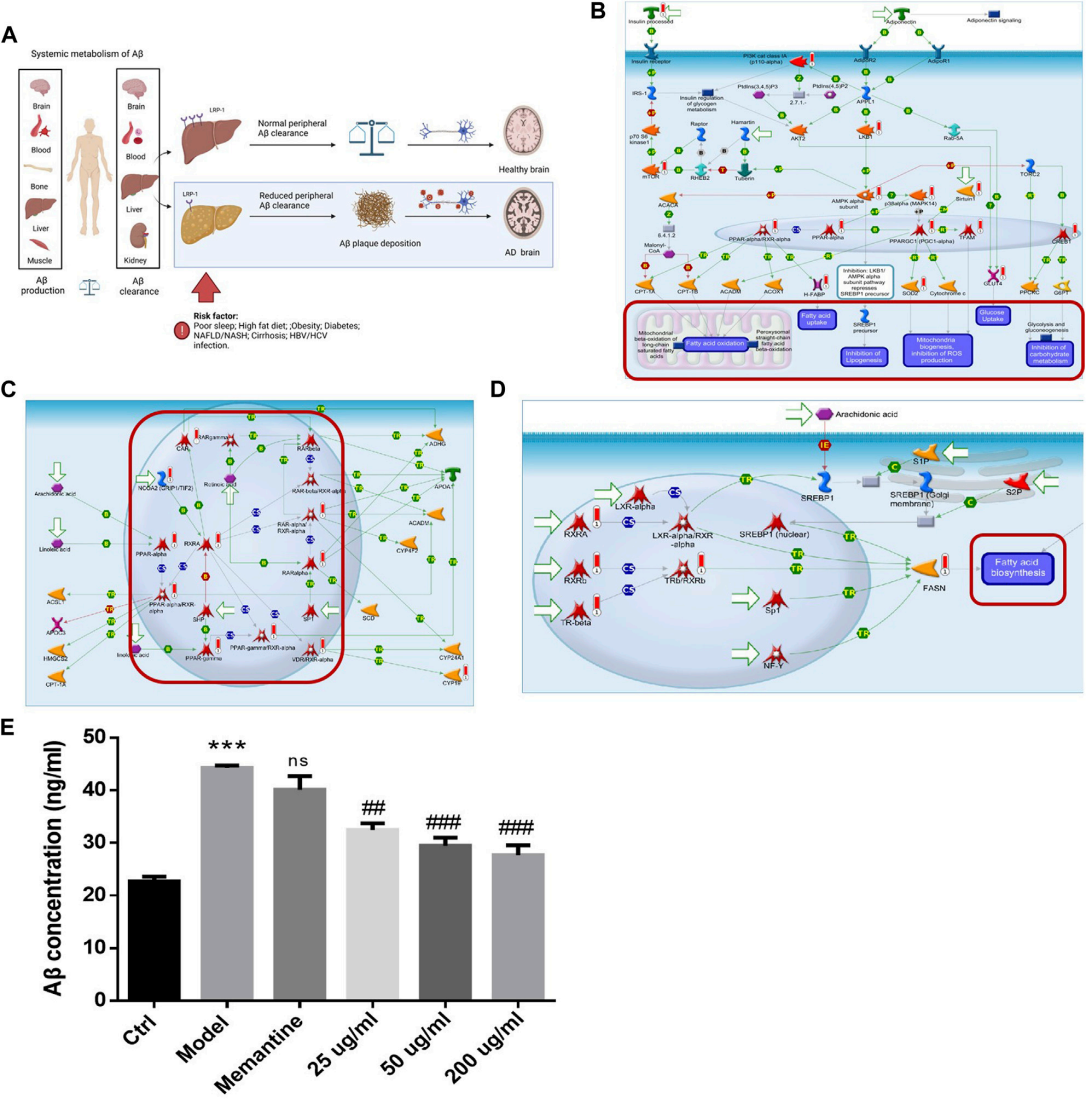
YXQNW promotes aβ degradation by regulating the glucose and lipid metabolisms in AD treatment. **(A)** Summary of liver dysfunction in AD; **(B)** regulation of the metabolism_role of Adiponectin in regulation of metabolism; **(C)** regulation of lipid metabolism_RXR-dependent regulation of lipid metabolism via PPAR, RAR, and VDR; **(D)** regulation of lipid metabolism_regulation of fatty acid synthase activity in hepatocytes. (The red marker is the target of YXQNW); **(E)** YXQNW may promote Aβ degradation in hepatic stellate cells. Rat hepatic stellate cell HST-T6 was incubated with different concentrations of YXQNW or negative control drug Memantine (100 μM). After 48 h of incubation, add Aβ1-42 (1 μM) into the cell except blank control group for 1 h of ingestion. Cellular Aβ accumulation was detected by an Aβ ELISA kit. ***p < 0.001 is compared with the Ctrl group; ##*p* < 0.01, ###*p* < 0.001 is compared with the Model group.

It is proved that Aβ deposits accumulate as plaques in the brain of an AD patient long before cognitive decline. Recently research has suggested that the liver is the origin of brain Aβ deposits and plays an essential role in the clearance of circulating Aβ (Bassendine et al., 2020). Chronic liver diseases with abnormal glucose and lipid metabolisms may increase the amyloid burden and promote Alzheimer’s pathological process (Figure 6A). Therefore, the liver could be targeted to decrease Aβ production or increase peripheral clearance. As mentioned previously, the YXQNW target set was analyzed for pathway enrichment in the MetaCore Database Analysis Platform. We focused on the pathway maps related to the regulation of glucose and lipid metabolisms in the liver. The results showed that YXQNW may regulate the glucose and lipid metabolisms in the liver through the following three pathways: 1) Regulation of the role of Adiponectin in the metabolism (Figure 6B); 2) regulation of lipid metabolism_RXR-dependent regulation of lipid metabolism via PPAR, RAR, and VDR (Figure 6C); and 3) regulation of lipid metabolism_regulation of fatty acid synthase activity in hepatocytes (Figure 6D). In addition, YXQNW may promote Aβ degradation by the regulation of selective insulin resistance in the liver (Data not shown).

In order to verify our hypothesis, we carried out experiments on rat hepatic stellate cell HST-T6 with Aβ_1-42_ incubation as described in the method. Compared with the model group, 0.025 mg/ml, 0.05 mg/ml, and 0.2 mg/ml YXQNW significantly decreased the cellular Aβ_1-42_ accumulation in the HST-T6 cell, but the negative control NMDA receptor antagonist memantine had no effect on Aβ_1-42_ accumulation. The concentration change trend (Figure 6E) indicated that YXQNW can promote the degradation of Aβ in hepatocytes, which further verified our previous hypothesis.

## 4 Discussion

Our study aimed to explore the mechanism of YXQNW in the treatment of AD. We proved that the overall correlation between YXQNW and AD is equivalent to clinical Western drugs, but the mechanism of action is very different. Firstly, YXQNW may promote cerebral blood flow velocity, which has been verified by clinical meta-analysis, by regulating platelet aggregation and the vasoconstriction/relaxation signal pathway. Secondly, YXQNW may promote Aβ degradation in the liver by modulating the abnormal glucose and lipid metabolism-related pathways, including the adiponectin-dependent pathway, RXR/PPAR-dependent lipid metabolism signal pathway, and fatty acid synthase activity signal pathway. We also verified that YXQNW indeed promotes Aβ degradation in hepatic stellate cells. This work provided a novel scientific basis for the key mechanism of YXQNW in the treatment of AD by “nourishing the blood and calming the liver.”

At present, the clinical first-line drugs for AD are directly targeted on te brain, such as the NMDA receptor antagonist (Memantine), AChE inhibitor (Donepezil, Galantamine, and Rivastigmine), and Serotonin and dopamine receptor antagonists (Quetiapine, Trifluperidol, and Risperidone). These drugs only alleviate some symptoms but do not rescue or delay the course of AD effectively. Now, most clinical trials begin at a relatively late stage of AD, when synaptic dysfunction, neuron loss, and cognitive impairment have already appeared. Many blockers against β/γ secretases, antibodies to the different forms of Aβ and blockers against tau protein phosphorylation have all failed in clinical trials (Masters, Bateman, Blennow, Rowe, Sperling and Cummings 2015; Kisler, Nelson, Montagne and Zlokovic 2017; Scheltens, De Strooper, Kivipelto, Holstege, Chetelat, Teunissen, Cummings and van der Flier 2021). Aducanumab antibody targeted to Aβ oligomers (Tolar et al., 2020) was approved by the FDA in 2021, but there is still great controversy about its effects of recovering cognitive impairment in AD patients. These research studies indicate that the treatment strategy of AD should be updated, especially looking for the targets in the early stages of AD.

Recently, more and more literature studies have reported that AD is not only a neurological disease, but is also accompanied by many peripheral and systemic abnormalities (Moon et al., 2011). AD is actually a systemic complex disease. There are research studies that suggest that the earliest event in AD is a decrease of the cerebral blood flow (CBF). Cerebral blood flow reduction was found in both AD patients, human-expressing ApoE4 protein which predisposes to AD, and AD mice models, which contributes to the pathological progression of AD (Thambisetty et al., 2010; Michels et al., 2016; Korte, Nortley and Attwell 2020). CBF reduction is probably caused by constriction of the capillaries by contractile pericytes (Nortley et al., 2019), neutrophil trapping in the capillaries (Hernandez et al., 2019), and clot formation (Cortes-Canteli et al., 2019). Through the HERB database, we determined that these targets related to the regulation of CBF mainly came from Dang Gui (Radix angelicae sinensis), Shu Di Huang (Radix rehmanniae preparata), Bai Shao (Radix paeoniae alba), Ji Xue Teng (Caulis spatholobi), and Jue Ming Zi (Semen cassiae). The literature reported that dabigatran, a direct oral anti-coagulant, preserved CBF and reduced the cognitive impairment in AD mice model (Cortes-Canteli, Kruyer, Fernandez-Nueda, Marcos-Diaz, Ceron, Richards, Jno-Charles, Rodriguez, Callejas, Norris, Sanchez-Gonzalez, Ruiz-Cabello, Ibanez, Strickland and Fuster 2019). More interestingly, the risk of dementia was reduced by 29% in humans who received oral anti-coagulants (Cerasuolo et al., 2017). Xueshuantong (XST), a TCM formula for increasing the blood flow in humans in China, can improve learning and memory and motor performance in APP/PS1 mice (Huang et al., 2018). These studies suggest that maintaining CBF may be an effective strategy for treatment of AD in the future. The effect of YXQNW on the improvement of CBF has been verified by many clinical experiments and mice models (Guo et al., 2019; Jia, Wang and Hou 2020). At the same time, YXQNW has also been proved to counteract the cognitive decline and decrease Aβ aggregation in the AD mouse model (Wang X et al., 2017). Our study first suggested the probable molecular mechanism of YXQNW on AD treatment by increasing CBF through the regulation of blood coagulation and the vasoconstriction/relaxation signal pathway.

Aβ is mostly generated in the brain (neurons, microglia, and astrocytes), blood (platelets), bone (osteoblasts), liver (hepatocyte), and skeletal muscle cells. About 40%–50% of brain-derived Aβ will be transported by carriers to peripheral tissues, where they are degraded or excreted via the liver or kidney (Wang J et al., 2017). The liver plays an essential role in the clearance of circulating Aβ (Masters, Bateman, Blennow, Rowe, Sperling and Cummings 2015; Kisler, Nelson, Montagne and Zlokovic 2017). Metabolic activities in the liver determine the state of peripheral Aβ circulation. A bulk of the evidence suggests that many metabolic disorders, including diabetes, obesity, and NASH, are risk factors for AD (De La Monte and Tong 2014; Kim et al., 2016; De La Monte 2017; Jin et al., 2017). Clinical studies have proved that altered liver function markers (serum AST to ALT ratio) are associated with AD pathophysiological characteristics (impaired memory, Aβ accumulation) (Peter 2004). This indicates that metabolic syndrome in the liver plays an important role in the pathophysiological charecteristics of AD. More evidence suggesting a new treatment strategy for AD may be targeted on the liver function to decrease Aβ production and increase peripheral clearance (Kisler, Nelson, Montagne and Zlokovic 2017). As a first-line lipid-lowering drug, atorvastatin has been shown to reduce AD risk possibility by upregulating liver LRP1 (Moon, Kang, Park, Lee, Kang, Ahn, Lee and Cha 2011; Zissimopoulos et al., 2017). Pioglitazone, used for the treatment of type 2 diabetes, has been proved to promote Aβ clearance in the mice model. Two large phase III clinical trials of Pioglitazone in AD are ongoing (Mandrekar-Colucci et al., 2012; Galimberti and Scarpini 2017). Silymarin, a medicinal herb for liver diseases, has been reported to reduce the production of Aβ oligomers and have potential for AD treatment (Murata et al., 2010). For the first time, our work proved that YXQNW can promote Aβ degradation in hepatocyte possibility by regulation of metabolism via adiponectin, PPAR/RAR, and fatty acid synthase activity (Figure 5). Adiponectin is an adipokine that sensitizes the insulin pathway and suppresses inflammation, which has been shown enhance the risk of AD (Kim et al., 2020). The PPAR (peroxisome promoter-activated receptor) family plays an important role in energy metabolism (Hong et al., 2019). PPARs agonist can significantly alleviate AD-related cognitive impairment in animal models, and relevant clinical trials are advancing (Wojtowicz et al., 2020). Through the HERB database, we determined that these targets related to regulating the liver function mainly come from Dang Gui (Radix angelicae sinensis), Jue Ming Zi (Semen cassiae), Yan Hu Suo (Rhizoma corydalis yanhusuo), Bai Shao (Radix paeoniae alba), Chuan Xiong (Rhizoma chuanxiong), and Gou Teng (Ramulus uncariae cum uncis). Among them, the key components targeting PPARs are palmitic acid from Dang Gui (Radix angelicae sinensis), emodin from Jue Ming Zi (Semen cassiae), and rutin from Chuan Xiong (Rhizoma chuanxiong). We first proved that YXQNW may play a role by improving the liver’s metabolic function in the treatment of AD through regulating multiple important metabolic-related pathways. Of course, the relationship between specific components and targets needs to be further verified.

It is interesting that the TCM theory of YXQNW is for “nourishing blood and calming liver”, which is consistent with our analysis results based on network pharmacology and machine learning. Although brain Aβ deposition, tau protein hyperphosphorylation, and neuroinflammation are still the most important targets for the treatment of AD, it is increasingly recognized that the systemic changes also contribute to the pathological progress of AD (Wang J et al., 2017). Traditional Chinese medicine is the typical multi-compound, multi-target, and multi-pathway agent (Wang X et al., 2017). Therefore, TCM provides a complementary and alternative approach to treat complex systemic diseases such as AD. This study firstly proved that YXQNW may alleviate related symptoms of AD patients by accelerating the blood flow velocity and regulating abnormal glucose and lipid metabolisms. More importantly, we verified our hypothesis by clinical meta-analysis and *in-vitro* Aβ accumulated experiments in hepatic stellate cells. However, this work still has some shortcomings. The effect of YXQNW on CBF and liver Aβ degradation were not verified in the AD model. In addition, relevant important signal pathways and targets of YXQNW were not directly determined. These works will be completed in the following work. In summary, different from the classical strategy of directly targeting the brain, this work provided a novel mechanism of YXQNW targeting the blood flow and liver function in the treatment of AD. Further study is necessary to study the relationship between the key compounds and important targets, which could provide more support for our hypothesis.

## Data Availability

The datasets presented in this study can be found in online repositories. The names of the repository/repositories and accession numbers can be found in the article/Supplementary Material.
