# Supplementary material for: Exploring the mechanism of YangXue QingNao Wan based on network pharmacology in the treatment of Alzheimer’s disease
Source: Front Genet. 2022 Aug 29;13:942203. doi: 10.3389/fgene.2022.942203 (PMC9465410; doi:10.3389/fgene.2022.942203)
Supplement: Supplementary file 1 [file DataSheet1.DOCX]

Supplementary Material

for

Exploring the mechanism of YangXue QingNao Wan based on network pharmacology in the treatment of Alzheimer's disease

Zhang Yuying ^1#^, Guo Kaimin ^1#^, Zhang Pengfei ^3^, Zhang Mengying ^1^, Li Xiaoqiang ^1^, Zhou Shuiping ^5, 6^, Sun He ^5, 6^, Wang Wenjia ^1^, Wang Hui ^2, 4,^* , and Hu Yunhui ^1^ *

^1^ Cloudphar Pharmaceuticals Co. Ltd., 518000, Shenzhen, China

^2^ Key Laboratory of Molecular Biophysics, Hebei Province, Institute of Biophysics, School of Health Sciences and Biomedical Engineering, Hebei University of Technology, Tianjin 300401, China

^3^ Tianjin Pharmaceutical and cosmetic evaluation and Inspection Center，Tianjin，300191，China

^4^ Key Laboratory of Bioactive Materials Ministry of Education, School of Life Sciences, Nankai University, 300071 Tianjin, China

^5^ The State Key Laboratory of Core Technology in Innovative Chinese Medicine, Tasly Academy, Tasly Holding Group Co., Ltd, 300410 Tianjin, China

^6^ Tasly Pharmaceutical Group Co., Ltd, 300410 Tianjin, China

**Running Title:** Mechanism of YXQNW in the treatment of AD

***** Correspondence: tsl-huyunhui@tasly.com; [autumnlop@163.com](mailto:autumnlop@163.com)

**#** YY Zhang and KM Guo contributed equally to this work

**Supporting results**

Table S1. Active ingredients of YXQNW.

| **Number** | **Ingredient Name** | **PubChemCID** | **Canonical SMILES** |
| --- | --- | --- | --- |
| 1 | Protocatechuic acid | 72 | C1=CC(=C(C=C1C(=O)O)O)O |
| 2 | Chlorogenic acid | 1794427 | C1C(C(C(CC1(C(=O)O)O)OC(=O)C=CC2=CC(=C(C=C2)O)O)O)O |
| 3 | Paeoniflorin | 442534 | CC12CC3(C4CC1(C4(C(O2)O3)COC(=O)C5=CC=CC=C5)OC6C(C(C(C(O6)CO)O)O)O)O |
| 4 | Caffeic acid | 689043 | C1=CC(=C(C=C1C=CC(=O)O)O)O |
| 5 | Gallic acid | 370 | C1=C(C=C(C(=C1O)O)O)C(=O)O |
| 6 | Ferulic acid | 445858 | COC1=C(C=CC(=C1)C=CC(=O)O)O |
| 7 | Rosmarinic acid | 5281792 | C1=CC(=C(C=C1CC(C(=O)O)OC(=O)C=CC2=CC(=C(C=C2)O)O)O)O |
| 8 | Tetrahydrocolumbamine | 440229 | COC1=C(C2=C(CC3C4=CC(=C(C=C4CCN3C2)OC)O)C=C1)OC |
| 9 | Corydaline | 101301 | CC1C2C3=CC(=C(C=C3CCN2CC4=C1C=CC(=C4OC)OC)OC)OC |
| 10 | α-Allocryptopine | 98570 | CN1CCC2=CC3=C(C=C2C(=O)CC4=C(C1)C(=C(C=C4)OC)OC)OCO3 |
| 11 | Tetrahydropalmatine | 5417 | COC1=C(C2=C(CC3C4=CC(=C(C=C4CCN3C2)OC)OC)C=C1)OC |
| 12 | Tetrahydroberberine | 34458 | COC1=C(C2=C(CC3C4=CC5=C(C=C4CCN3C2)OCO5)C=C1)OC |
| 13 | Dehydrocorydaline | 34781 | CC1=C2C=CC(=C(C2=C[N+]3=C1C4=CC(=C(C=C4CC3)OC)OC)OC)OC |
| 14 | Tetrahydrocoptisine | 6770 | C1CN2CC3=C(CC2C4=CC5=C(C=C41)OCO5)C=CC6=C3OCO6 |
| 15 | Quinic acid | 6508 | C1C(C(C(CC1(C(=O)O)O)O)O)O |
| 16 | Ligustilide | 5877292 | CCCC=C1C2=C(C=CCC2)C(=O)O1 |
| 17 | Palmitic acid | 985 | CCCCCCCCCCCCCCCC(=O)O |
| 18 | Z-Ligustilide | 5319022 | CCCC=C1C2=C(C=CCC2)C(=O)O1 |
| 19 | Acteoside | 5281800 | CC1C(C(C(C(O1)OC2C(C(OC(C2OC(=O)C=CC3=CC(=C(C=C3)O)O)CO)OCCC4=CC(=C(C=C4)O)O)O)O)O)O |
| 20 | Aloe-emodin | 10207 | C1=CC2=C(C(=C1)O)C(=O)C3=C(C2=O)C=C(C=C3O)CO |
| 21 | Catechin | 9064 | C1C(C(OC2=CC(=CC(=C21)O)O)C3=CC(=C(C=C3)O)O)O |
| 22 | Catechinic acid | 9064 | C1C(C(OC2=CC(=CC(=C21)O)O)C3=CC(=C(C=C3)O)O)O |
| 23 | Chrysoobtusin | 155381 | CC1=CC2=C(C(=C1O)OC)C(=O)C3=C(C(=C(C=C3C2=O)OC)OC)OC |
| 24 | Chrysophanol | 10208 | CC1=CC2=C(C(=C1)O)C(=O)C3=C(C2=O)C=CC=C3O |
| 25 | Emodin | 3220 | CC1=CC2=C(C(=C1)O)C(=O)C3=C(C2=O)C=C(C=C3O)O |
| 26 | Epicatechin | 72276 | C1C(C(OC2=CC(=CC(=C21)O)O)C3=CC(=C(C=C3)O)O)O |
| 27 | Hirsutine | 3037884 | CCC1CN2CCC3=C(C2CC1C(=COC)C(=O)OC)NC4=CC=CC=C34 |
| 28 | Hyperoside | 5281643 | C1=CC(=C(C=C1C2=C(C(=O)C3=C(C=C(C=C3O2)O)O)OC4C(C(C(C(O4)CO)O)O)O)O)O |
| 29 | Mitraphylline | 94160 | CC1C2CN3CCC4(C3CC2C(=CO1)C(=O)OC)C5=CC=CC=C5NC4=O |
| 30 | Physcion | 10639 | CC1=CC2=C(C(=C1)O)C(=O)C3=C(C2=O)C=C(C=C3O)OC |
| 31 | Protocatechualdehyde | 8768 | C1=CC(=C(C=C1C=O)O)O |
| 32 | Pteropodine | 10429112 | CC1C2CN3CCC4(C3CC2C(=CO1)C(=O)OC)C5=CC=CC=C5NC4=O |
| 33 | Rutin | 5280805 | CC1C(C(C(C(O1)OCC2C(C(C(C(O2)OC3=C(OC4=CC(=CC(=C4C3=O)O)O)C5=CC(=C(C=C5)O)O)O)O)O)O)O)O |
| 34 | Vanillic acid | 8468 | COC1=C(C=CC(=C1)C(=O)O)O |
| 35 | Umbelliferone | 5281426 | C1=CC(=CC2=C1C=CC(=O)O2)O |

Table S2 The efficacy of YXQNW on related indications by network analysis.

| **Drug** | **Number of Targets** | **Indication** | **Number of Genes** | **Original coef** | **Random coef** | **Z-score** |
| --- | --- | --- | --- | --- | --- | --- |
| YXQNW | 404 | Alzheimer’s Disease | 468 | 0.2766367 | 0.01049503 | 19.60656 |
| YXQNW | 404 | Vascular Dementia | 156 | 0.287411 | 0.006869 | 19.29932 |
| YXQNW | 404 | Hypertensive Encephalopathy | 177 | 0.227031 | 0.006388 | 15.83864 |
| YXQNW | 404 | Hypertension | 328 | 0.213406 | 0.008923 | 14.58552 |
| YXQNW | 404 | Cerebral small vessel diseases | 497 | 0.181599 | 0.011779 | 12.34755 |
| YXQNW | 404 | Prehypertension | 86 | 0.149528 | 0.004289 | 10.07693 |
| YXQNW | 404 | Vertebrobasilar Insufficiency | 29 | 0.069001 | 0.002197 | 4.774695 |

Table S3 Profiles of transcriptomics datasets for Alzheimer‘s disease from 3 of different brain regions of transgenic mouse models

| Dataset | ORGANISM | GENETIC BACKGROUND | MODEL | AGE | REGION | GROUP | CRITERIA | GENES | GEO |
| --- | --- | --- | --- | --- | --- | --- | --- | --- | --- |
| D1 | Mouse | C57Bl/6J | HO-TASTPM | 8 months | Hippocampus | 4 AD mice vs. 9 controls | FDR < 0.05, \|FC\| > 1.5 | 211 up / 32 down | GSE64398 |
| D2 | Mouse | C57Bl/6J | HO-TASTPM | 18 months | Hippocampus | 4 AD mice vs. 9 controls | FDR < 0.05, \|FC\| > 1.5 | 258 up / 45 down | GSE64398 |
| D3 | Mouse | C57Bl/6J | HO-TASTPM | 8 months | Frontal cortex | 4 AD mice vs. 9 controls | FDR < 0.05, \|FC\| > 1.5 | 135 up / 1 down | GSE64398 |
| D4 | Mouse | C57Bl/6J | HO-TASTPM | 18 months | Frontal cortex | 3 AD mice vs. 7 controls | FDR < 0.05, \|FC\| > 1.5 | 234 up / 19 down | GSE64398 |
| D5 | Mouse | C57Bl/6J | APP/PS1 | 8 months | Brain | 5 AD mice vs. 5 controls | FDR < 0.05, \|FC\| > 1.5 | 513 up / 344 down | GSE65067 |
| D6 | Mouse | A cross between C57BL/6J and C3H/HeJ | APP/PS1 | 15-18 months | Brain | 7 AD mice vs. 7 controls | FDR < 0.05, \|FC\| > 1.5 | 277 up / 477 down | GSE74615 |

Table S4 Evaluation of efficacy of YXQNW on Alzheimer’s disease using differential expression genes by network analysis

| **Drug** | **Number of Targets** | **AD-related Dataset** | **Number of Genes** | **Original coef** | **Random coef** | **Z-score** |
| --- | --- | --- | --- | --- | --- | --- |
| YXQNW | 404 | D1 | 243 | 0.066506 | 0.007449 | 4.342017 |
| YXQNW | 404 | D2 | 303 | 0.096995 | 0.009133 | 6.331495 |
| YXQNW | 404 | D3 | 136 | 0.081365 | 0.00625 | 5.333815 |
| YXQNW | 404 | D4 | 253 | 0.085914 | 0.008618 | 5.552559 |
| YXQNW | 404 | D5 | 857 | 0.10723 | 0.015409 | 6.552526 |
| YXQNW | 404 | D6 | 754 | 0.099489 | 0.015096 | 6.005505 |

Table S5 The efficacy of YXQNW and Clinical Western Medicine on Alzheimer’s disease by network analysis.

| **Drug** | **Number of Targets** | **Disease** | **Number of Genes** | **Original coef** | **Random coef** | **Z-score** |
| --- | --- | --- | --- | --- | --- | --- |
| YXQN | 404 | AD | 468 | 0.2766367 | 0.01049503 | 19.60656 |
| Haloperidol | 326 | AD | 468 | 0.21485093 | 0.009259215 | 13.44567 |
| Donepezil | 164 | AD | 468 | 0.208091739 | 0.006443281 | 13.01235 |
| Memantine | 309 | AD | 468 | 0.221714177 | 0.010399695 | 12.34474 |
| Risperidone | 399 | AD | 468 | 0.180924896 | 0.009924102 | 12.23467 |
| Trifluperidol | 50 | AD | 468 | 0.145117613 | 0.001402846 | 11.84958 |
| Galantamine | 173 | AD | 468 | 0.181529297 | 0.006270802 | 11.47656 |
| Piracetam | 121 | AD | 468 | 0.169895078 | 0.003179847 | 10.3257 |
| Quetiapine | 210 | AD | 468 | 0.154733193 | 0.003211445 | 10.07727 |
| Rivastigmine | 63 | AD | 468 | 0.194295861 | 0.003502928 | 9.905071 |
| Citalopram | 118 | AD | 468 | 0.138790258 | 0.004136104 | 9.442818 |
| Vinpocetine | 54 | AD | 468 | 0.12059947 | 0.002117871 | 9.087052 |
| Idebenone | 84 | AD | 468 | 0.083224549 | 0.004122045 | 5.240801 |
| Pegvaliase  (negtive control) | 45 | AD | 468 | 0.044983871 | 0.005438575 | 2.675867 |
| Sapropterin  (negtive control) | 45 | AD | 468 | 0.044983871 | 0.00570932 | 2.468104 |


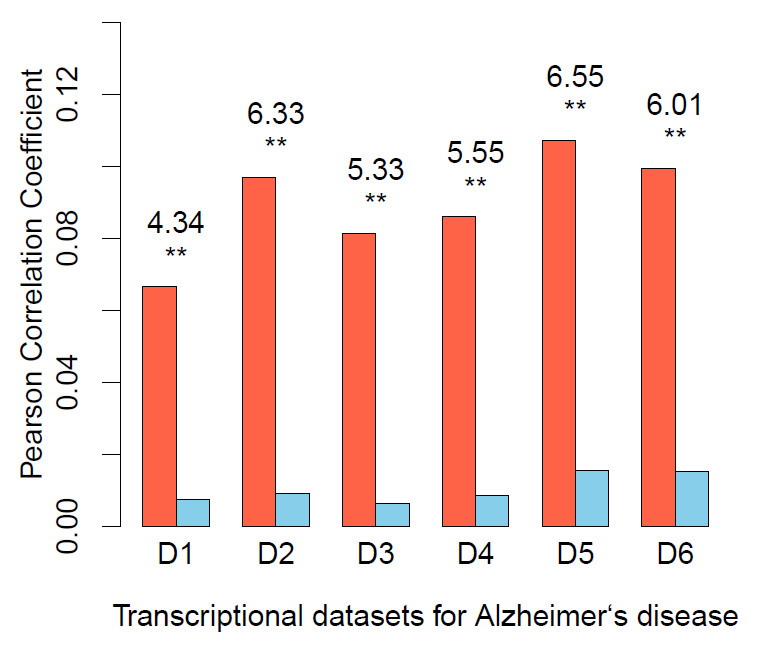
 Figure S1. Evaluation of correlation of YXQNW with Alzheimer’s disease using different transcriptional dataset


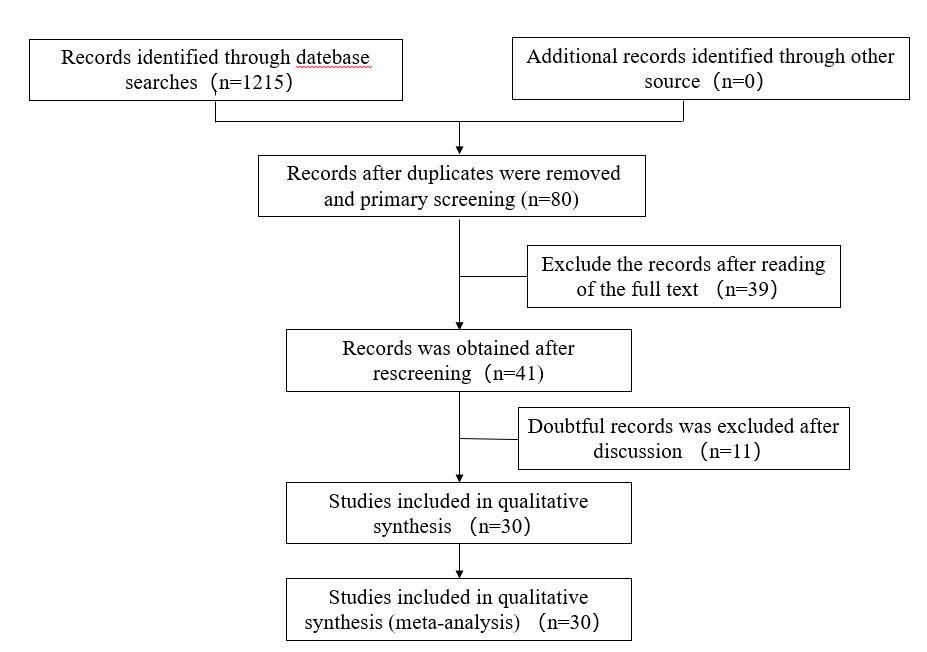


Figure S2. Screening process of literature


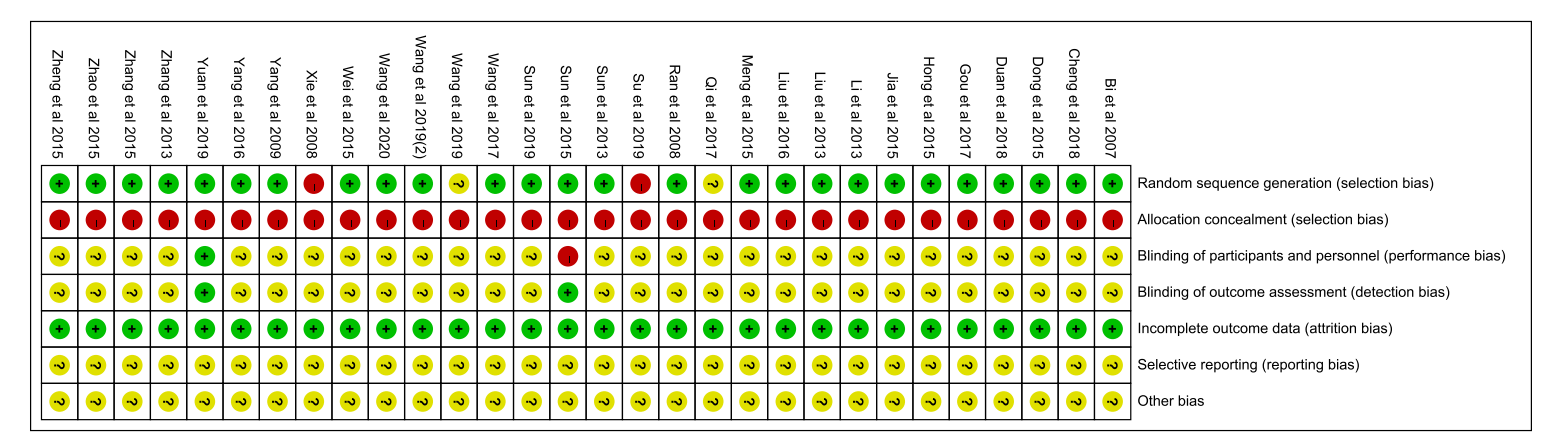


Figure S3. Bias risk graph


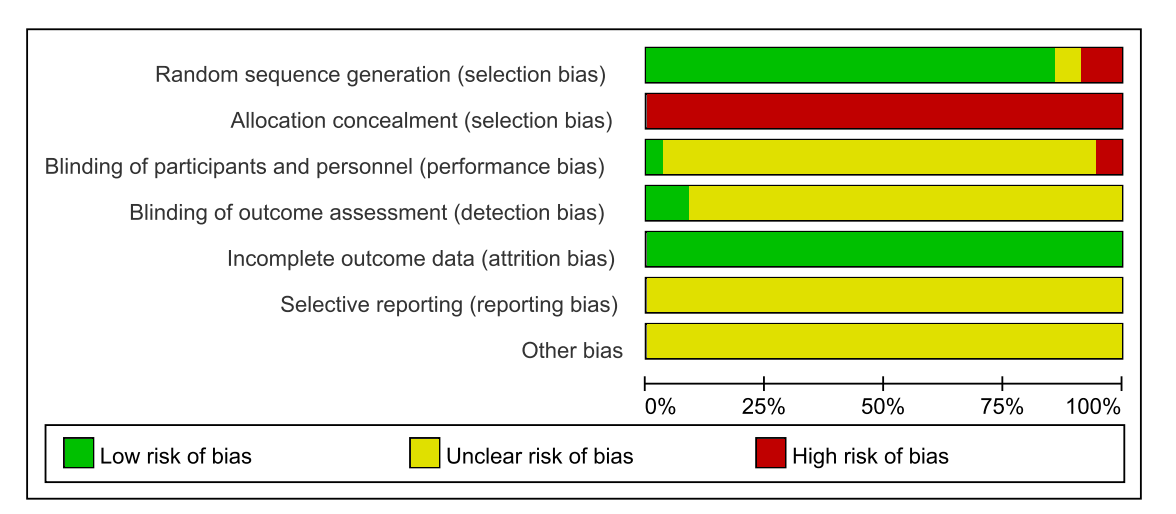


Figure S4. Bar graph of bias risk


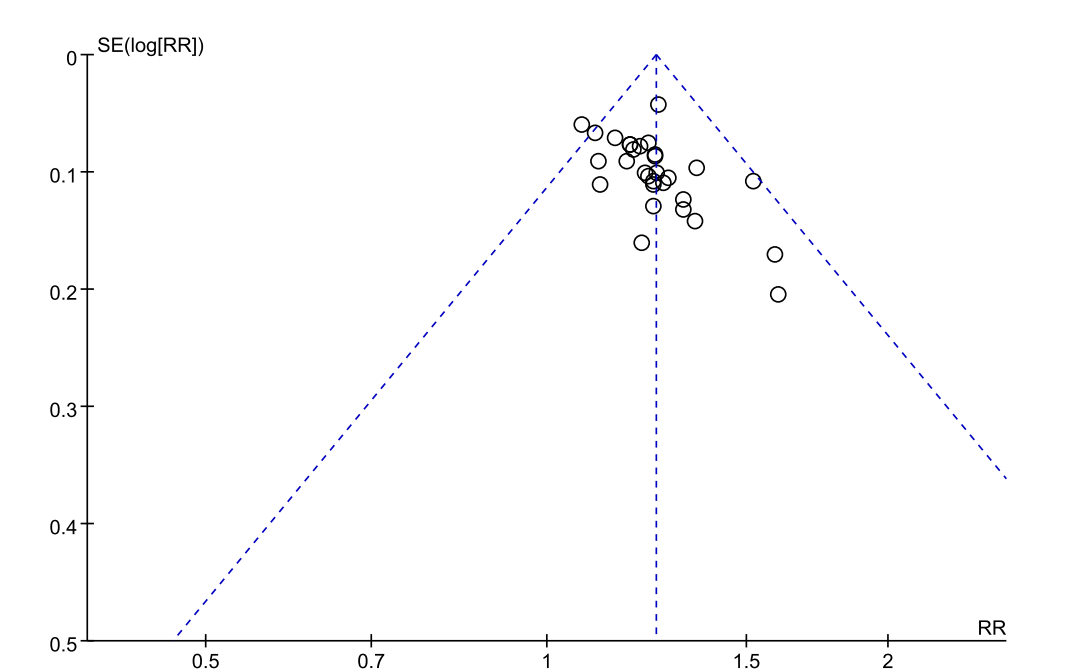


Figure S5. Inverted funnel plot of clinical efficacy
